# Skin carotenoids indicate diet, serum carotenoids, and inflammation across obesity and metabolic status in children

**DOI:** 10.1186/s12986-026-01075-7

**Published:** 2026-02-22

**Authors:** Yang Liu, Huihui Huang, Chi Sun, Wenhan Jia, Xuxiu Zhuang, Jia Zheng, Le Jiang, Yanan Ma, Bing Song, Joel Gittelsohn, Deliang Wen

**Affiliations:** 1 Health Sciences Institute, China Medical University, No.77 Puhe Road, Shenyang North New Area, Shenyang, 110122 Liaoning China; 2 Liaoning Key Laboratory of Obesity and Glycolipid Metabolic Diseases, Puhe Rd.77, Shenyang, 110122 Liaoning China; 3 Experiment Center of Science, China Medical University, Puhe Rd.77, Shenyang, 110122 Liaoning China; 4 School of Public Health, China Medical University, Puhe Rd.77, Shenyang, 110122 Liaoning China; 5 First Affiliated Hospital of Jinzhou Medical University, 2-5 Renmin Road, Jinzhou, 121001 China; 6 Department of International Health, Human Nutrition Center, Johns Hopkins Bloomberg School of Public Health, 615 North Wolfe St Baltimore, Baltimore, MD 21205-2179 USA

**Keywords:** Skin carotenoids, Obesity, Metabolic dysfunction, Fruit and vegetable intake, Biomarkers

## Abstract

**Objectives:**

To validate reflection spectroscopy (RS)-based skin carotenoids (SCS) as non-invasive biomarkers of fruit and vegetable intake (FVI) in children and adolescents with obesity and metabolic dysfunction.

**Methods:**

This case-control study (China, 2023) included 210 children and adolescents aged 7–17 years, categorized into five groups: healthy weight (*n* = 30), overweight (*n* = 23), obesity (*n* = 25), obesity with one metabolic disorder (*n* = 56), and obesity with two or more metabolic disorders (*n* = 76). SCS levels were measured using RS, FVI was assessed via a food frequency questionnaire, serum carotenoids were quantified by high-performance liquid chromatography, and inflammatory markers were analyzed using flow cytometry.

**Results:**

SCS and serum carotenoid levels decreased significantly across groups (p for trend < 0.001). SCS correlated strongly with serum carotenoids, except for lycopene, with the strongest association observed in children and adolescents with obesity and two or more metabolic disorders. A quartile increase in FVI resulted in an 11.92–45.98 unit increase in SCS, partially mediated by total serum carotenoids, beta-carotene, and beta-cryptoxanthin. SCS also inversely correlated with inflammation in metabolically disordered groups.

**Conclusions:**

RS-based SCS is a valid, non-invasive biomarker of FVI, closely related to serum carotenoids and inflammatory status, and effectively distinguishes metabolic dysfunction in children and adolescents.

**Supplementary Information:**

The online version contains supplementary material available at 10.1186/s12986-026-01075-7.

## Introduction

Since 1980, global obesity rates have steadily risen. The prevalence obesity among children and adolescents has dramatically increased, with the age-standardized rates for girls and boys reaching 6.9% and 9.3%, respectively, by 2022 [1, 2]. A healthy diet rich in fruits and vegetables is essential for maintaining a healthy weight, yet many adolescents fall short of dietary guidelines [3, 4]. For example, only 14.2% of Chinese children and adolescents meet the recommended vegetable intake, and just 3.1% meet the fruit intake guideline [5]. Thus, improving children and adolescents’ fruit and vegetable intake (FVI) has become a key objective of public health interventions worldwide.

Accurately measuring FVI in children and adolescents is challenging. Common methods, such as caregiver reports or self-reports, are prone to biases, including recall and social desirability bias [6–10]. Observational methods and plate waste analysis also have limitations. Biomarkers offer a promising alternative [11]. Carotenoids, natural pigments found in fruits and vegetables, are recognized as biomarkers for FVI [10, 12]. Skin carotenoids scores (SCS), measured noninvasively using devices like the Veggie Meter^®^ (VM^®^), provide a reliable indicator of FVI [13–16]. Although VM^®^ has been validated in adults [17–23], research on its validity in children and adolescents [24–30], especially against serum carotenoid levels, remains limited.

Carotenoids also possess anti-inflammatory properties relevant to obesity-related inflammation. Studies have shown correlation between SCS and body mass index (BMI) or MetS [15, 26, 31]. However, evidence on the use of SCS to differentiate weight status groups and their relationship with inflammatory markers is scarce. This study aims to assess the validity of VM^®^-measured SCS in children and adolescents with varying weight statuses and metabolic disorders. Additionally, it examines associations between SCS, FVI, serum carotenoid levels, and inflammatory markers.

## Methods

### Study design and participants

This case-control study utilized data from children and adolescents aged 7–17 years who participated in the Family Cohort of Obesity-Related Chronic Diseases (FC-OCD) in Liaoning, China, in 2023. The FC-OCD is an ongoing longitudinal study initiated in 2022. Participants were recruited via announcements distributed through school networks. The cohort focuses on family units, enrolling children, their parents, and siblings. Although a key recruitment emphasis was placed on identifying overweight or obese children, the study also welcomed the participation of normal-weight children and their families based on interest. As of the current data cut-off, a total of 2122 families have been successfully recruited. Both cases and controls were drawn from the baseline FC-OCD, including children and adolescents in the following categories (Figure S1): healthy weight (HW) (*n* = 30), overweight (OW) (*n* = 23), obesity (OB) (*n* = 25), obesity with one metabolic disorder (OB & one MD) (*n* = 56), and obesity with two or more metabolic disorders (OB & two or more MDs) (*n* = 76). Metabolic disorders were defined as abnormalities in blood pressure, blood glucose, blood lipids, and uric acid as follows:

Blood pressure (BP) was classified into three categories: normal, high-normal, and high blood pressure. **High-normal blood pressure** is defined as systolic blood pressure (SBP) and/or diastolic blood pressure (DBP) that is greater than or equal to the 90th percentile for individuals of the same sex, age, and height, but less than the 95th percentile. It also includes cases where SBP is ≥ 120 mmHg (but below the 95th percentile for individuals of the same sex, age, and height) and/or DBP is ≥ 80 mmHg (but below the 95th percentile for individuals of the same sex, age, and height). **High blood pressure** is defined as SBP and/or DBP that is greater than or equal to the 95th percentile for individuals of the same sex, age, and height [32]. Participants with either high-normal or high blood pressure were classified as having a blood pressure disorder.

Impaired FPG: Participants were classified as having a blood glucose disorder if their fasting serum glucose was greater than or equal to 5.6 mmol/L [33].

Dyslipidemia: Participants were classified as having a blood lipid disorder if their cholesterol (CHOL) was greater than or equal to 5.18 mmol/L, low-density lipoprotein (LDL) was greater than or equal to 3.37 mmol/L, triglycerides (TG) were greater than or equal to 1.70 mmol/L, or high-density lipoprotein (HDL) was less than or equal to 1.04 mmol/L [34].

Hyperuricemia (HUA): The criteria for diagnosing hyperuricemia are as follows: uric acid levels > 320 µmol/L for children aged 1–10, > 470 µmol/L for boys aged 11–15, > 350 µmol/L for girls aged 11–15, and > 420 µmol/L for individuals over 15, following the adult standard [35].

Overweight and obesity were defined according to cut-off BMI recommended by Working Group on Obesity in China (WGOC), as P85 and P95 respectivley, based on data collection by the Chinese National Survey on Students Constitution and Health [36]. Signed informed consent was obtained from the participating children and their parents or legal guardians. The study was approved by Ethics Committee of China Medical University (Approval No: Ethics Review [2021] 124).

### Data collection

#### Physical examination

*Height*,* weight and body fat* Height was measured by Seca213 portable stadiometer, weight and fat percentage were measured with Tanita DC-13 body composition analyzer. The average of two measurements was taken.

*Blood pressure (BP)* BP was measured with the subject at rest and in a sitting position using an Omron medical blood pressure monitor with an appropriately sized cuff. Three measurements were taken 5 min apart and the average value was used in the analysis for systolic BP (SBP) and diastolic BP (DBP).

#### SCS measurement

SCS were measured by trained research staff using a reflection spectroscopy device (VeggieMeter, Longevity Link Co.), with its operational principles detailed elsewhere [37]. The device minimizes the impact of blood perfusion by applying pressure to the fingertip and measures skin carotenoid levels with minimal interference from melanin pigmentation, producing scores from 0 to 800. Measurements were conducted per the manufacturer’s guidelines. Calibration with reference materials provided by the manufacturer was performed daily before measurements, which took place twice daily (prior to the morning and afternoon sessions). For each measurement, participants placed their left ring finger in the device’s cradle. The skin carotenoid index was calculated as the average of three consecutive measurements for each participant.

#### FVI measurement

FVI was assessed using a food frequency questionnaire (FFQ) completed by children and adolescents in the presence of their caregivers and interviewers. Validated FFQ of China Health and Nutrition Survey was used and adapted by adding local food items [38]. During the interview, a validated handout with images illustrating various food portion sizes, represented by common objects, was provided [39]. Children and adolescents and caregivers were asked to recall, over the past year, how frequently each food type was consumed (options included: never, less than once per month, 1–3 times per month, 1–2 times per week, 3–4 times per week, 5–6 times per week, once per day, twice per day, or more than three times per day) and the average portion size per instance. The questionnaire included 22 types of common dietary items, such as cereals, meats, seafood, dairy, snacks, beverages, vegetables, and fruits. The average daily FVI (g) was calculated for each child.

#### Serum carotenoid measurement

Serum carotenoid concentration was measured using high-performance liquid chromatography (Thermo DGLC diatomic ultra high-performance liquid phase system). Quantification was done using an external standard method. A calibration curve was prepared using different concentrations of standard samples to determine the concentrations of beta-carotene, alpha-carotene, lycopene, beta-cryptoxanthin, lutein, and zeaxanthin.

### Metabolic indicators

Fasting blood glucose, triglycerides (TG), total cholesterol (T-CHO), high-density lipoprotein cholesterol (HDL-C), low-density lipoprotein cholesterol (LDL-C), and uric acid (UA) were measured in hospital laboratories using standard enzymatic assays. Blood samples were collected using EDTA-coated vacuum blood collection tube after an overnight fast of at least 8 h. Fasting blood glucose was assessed by a glucose oxidase-peroxidase method. TG, T-CHO, HDL-C, and LDL-C levels were measured using enzymatic colorimetric methods, while uric acid concentration was determined through a uricase-based enzymatic assay. These assays were conducted using automated biochemical analyzers (Abbott ACCELERATOR a3600) following the manufacturers’ protocols to ensure consistency and accuracy.

### Serum inflammatory cytokines

Inflammatory cytokines, including IL-1β, IL-6, IL-13, IL-4, IL-10, MCP-1, IL-2, IL-12p70, IL-23p19, TNF-α, and IFN-γ, were measured using a flow cytometry analyzer (BD FACSCanto™ Flow Cytometer). A 25 µL diluted serum sample was prepared and loaded through an automated high-throughput sampler. Final results were analyzed using BD FACSDiva software.

### Covariates

Age, sex, and carotenoid supplement intake were included as covariates. Age and sex information was collected through questionnaires completed by children and adolescents and their caregivers before the physical examination. Caregivers were also asked if their child had taken any dietary supplements, such as lutein or carotenoids, within the past year, prior to the SCS measurement and on the day of the physical examination.

### Statistical analysis

**Fig. 1:**
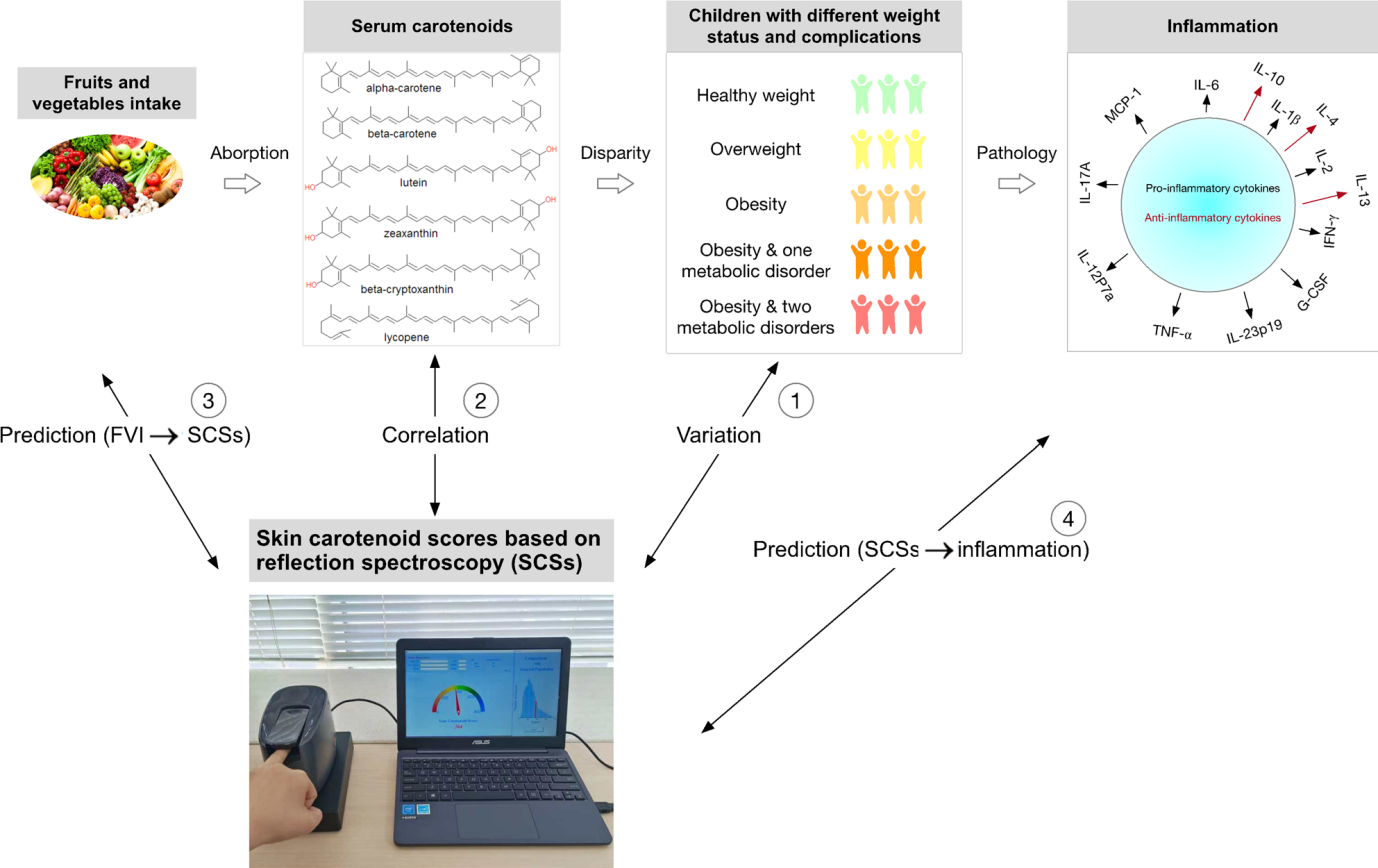
Diagram of the conceptual model of the analysis of study results

All analyses were conducted using R version 4.4.1. The threshold for statistical significance was set at a p-value of < 0.05 for all two-tailed tests. Prior to analysis, the normality of the distribution for all continuous variables was assessed using the Shapiro-Wilk test. Based on these results, baseline characteristics were summarized and compared across the five obesity and metabolic disorder groups using ANOVA, Kruskal-Wallis test, chi-square test, or Fisher’s exact test, as appropriate, with the *CreateTableOne* function from the *tableone* package in R. As shown in Fig. 1, the analysis was divided into five parts.

First, a violin plot with a boxplot overlay was used to illustrate the variation in SCS and serum carotenoids across the five groups, with trends tested using the Jonckheere-Terpstra test. The *jonckheere.test* function from the *clinfun* package and the *ggplot2* package were used for this analysis.

Second, Spearman correlation coefficients between serum carotenoid levels and SCS were calculated using the *cor.test* function from the *stats* package. The *ggplot2* package was used to create a heatmap to visualize the correlations. The analysis was stratified by obesity and metabolic disorder groups.

Third, *β* coefficients (95% *CI*) for the change in SCS associated with a one-unit increase in FVI quartile were calculated using linear regression (ordinary least squares) with the *lm* function from the base R package. Mediation analysis was conducted to assess the role of serum carotenoids in the association between FVI and SCS, calculating the proportion of the total effect of FVI quartiles on SCS explained by serum carotenoids. This analysis was performed using the *mediate* function from the *mediation* package. The analysis was stratified by obesity and metabolic disorder groups, with age, sex, and carotenoid supplement intake included as covariates.

Fourth, *β* coefficients (95% *CI*) for the change in each inflammatory marker associated with a one-unit increase in SCS tertile were calculated using linear regression (ordinary least squares) with the *lm* function from the base R package. The *ggplot2* package was used to create a forest plot of the regression estimates and their 95% confidence intervals for each dependent variable. The analysis was stratified by obesity and metabolic disorder groups, with age, sex, and BMI included as covariates.

For part two, part three, and part four, missing values in the dataset are imputed using K-Nearest Neighbors (KNN) Imputation based on the values of the 5 nearest neighbors (k = 5), filling gaps in the data by using similar observations.

## Results

### Characteristics of the study participants

The main characteristics of participants across the BMI groups are presented in Table 1. The mean age of all participants is 12.42 ± 2.87 years. As hypothesized, BMI, SBP, DBP, TG, LDL, and UA levels differ significantly among the groups (*p* < 0.001 of *p* = 0.001). These measures were highest in the “OB & Two or more MDs” group and lowest in the “HW” group, while the opposite trend was observed for HDL. In contrast, differences in FPG and total cholesterol (CHOL) between the groups are not significant. The groups also show significant differences in daily fruit and vegetable intake, with “HW” group recording the highest consumption (*p* = 0.002). However, there is no significant difference in supplement intake among the groups (*p* > 0.05).

**Table 1:** Characteristics of participants by BMI groups

Characteristics	HW (*n* = 30)		OW (*n* = 23)	OB (*n* = 25)	OB & One MD (*n* = 56)	OB & Two or more MDs (*n* = 76)	*P*- value
**Basic information**							
Age, *years*	12.03 (2.74)		12.04 (2.16)	12.72 (2.32)	11.75 (3.05)	13.55 (2.66)	0.002
Sex, *n (%) female*	18 (60)		14 (61)	19 (76)	27 (48)	17 (22)	< 0.001
**Physical examination**							
BMI, *kg/m*^*2*^	18.17 (2.40)		22.53 (2.12)	27.70 (3.83)	26.84 (4.74)	29.39 (4.13)	< 0.001
Weight status, *n (%)*							< 0.001
Normal	30 (100)		0 (0)	0 (0)	0 (0)	0 (0)	
Overweight	0 (0)		23 (100)	0 (0)	0 (0)	0 (0)	
Obesity	0 (0)		0 (0)	25 (100)	56 (100)	76 (100)	
SBP, *mmHg*	104.13 (7.52)		107.13 (8.64)	106.92 (8.80)	115.71 (15.09)	125.54 (13.27)	< 0.001
DBP, *mmHg*	59.90 (5.98)		58.30 (6.74)	58.96 (6.72)	62.96 (12.63)	66.47 (10.20)	< 0.001
High (normal) BP, *n (%)*	0 (0)		0 (0)	0 (0)	23 (41)	56 (74)	< 0.001
**Metabolic indicators**							
FPG, *mmol/L*	4.74 (0.32)		4.84 (0.36)	4.76 (0.42)	4.71 (0.36)	4.85 (0.62)	0.474
Impaired FPG, *n (%)*	0 (0)		0 (0)	0 (0)	0 (0)	4 (5)	0.700
CHOL, *mmol/L*	3.65 (0.54)		3.72 (0.47)	3.81 (0.45)	3.92 (0.74)	3.89 (1.05)	0.547
TG, *mmol/L*	0.62 (0.17)		0.80 (0.29)	0.93 (0.29)	0.98 (0.45)	1.21 (0.59)	< 0.001
LDL, *mmol/L*	1.99 (0.45)		2.17 (0.46)	2.27 (0.34)	2.39 (0.60)	2.55 (0.88)	0.001
HDL, *mmol/L*	1.44 (0.31)		1.30 (0.15)	1.30 (0.17)	1.28 (0.26)	1.09 (0.19)	< 0.001
Dyslipidemia, *n (%)*	0 (0)		0 (0)	0 (0)	12 (21)	49 (64)	< 0.001
UA, *µmol/L*	306.14 (47.32)		346.02 (67.30)	354.90 (79.78)	403.16 (107.12)	474.12 (90.00)	< 0.001
HUA, *n (%)*	0 (0)		0 (0)	0 (0)	21 (38)	61 (80)	0.001
**FVI measurement**							
FVI, *g/day*	116.36 (163.20)		57.53 (97.17)	24.08 (65.38)	25.26 (55.34)	45.19 (110.77)	0.002
Supplement intake, *n (%) Yes*		9 (30)	6 (26)	5 (20)	12 (21)	9 (12)	0.200

Values are means (SDs) or n (%). ANOVA was used for BMI, SBP, DBP, FPG, CHOL, TG, LDL, HDL, and UA. Kruskal-Wallis test was used for age and FVI. Chi-square test was used for sex and supplement intake. Fisher’s exact test was used for weight status, high BP, impaired FPG, dyslipidemia and HUA

Abbreviations: HW, healthy weight; OW, overweight; OB, obesity; MD, metabolic disorder; BMI: body mass index; SBP: systolic blood pressure; DBP: diastolic blood pressure; High (normal) BP: high normal blood pressure or high blood pressure; FPG: fasting serum glucose; CHOL: cholesterol; TG: triglyceride; LDL: low density lipoprotein; HDL: high density lipoprotein; UA: uric acid; HUA: Hyperuricemia; FVI: fruit and vegetable intake

### Variation in carotenoids across BMI groups

**Fig. 2:**
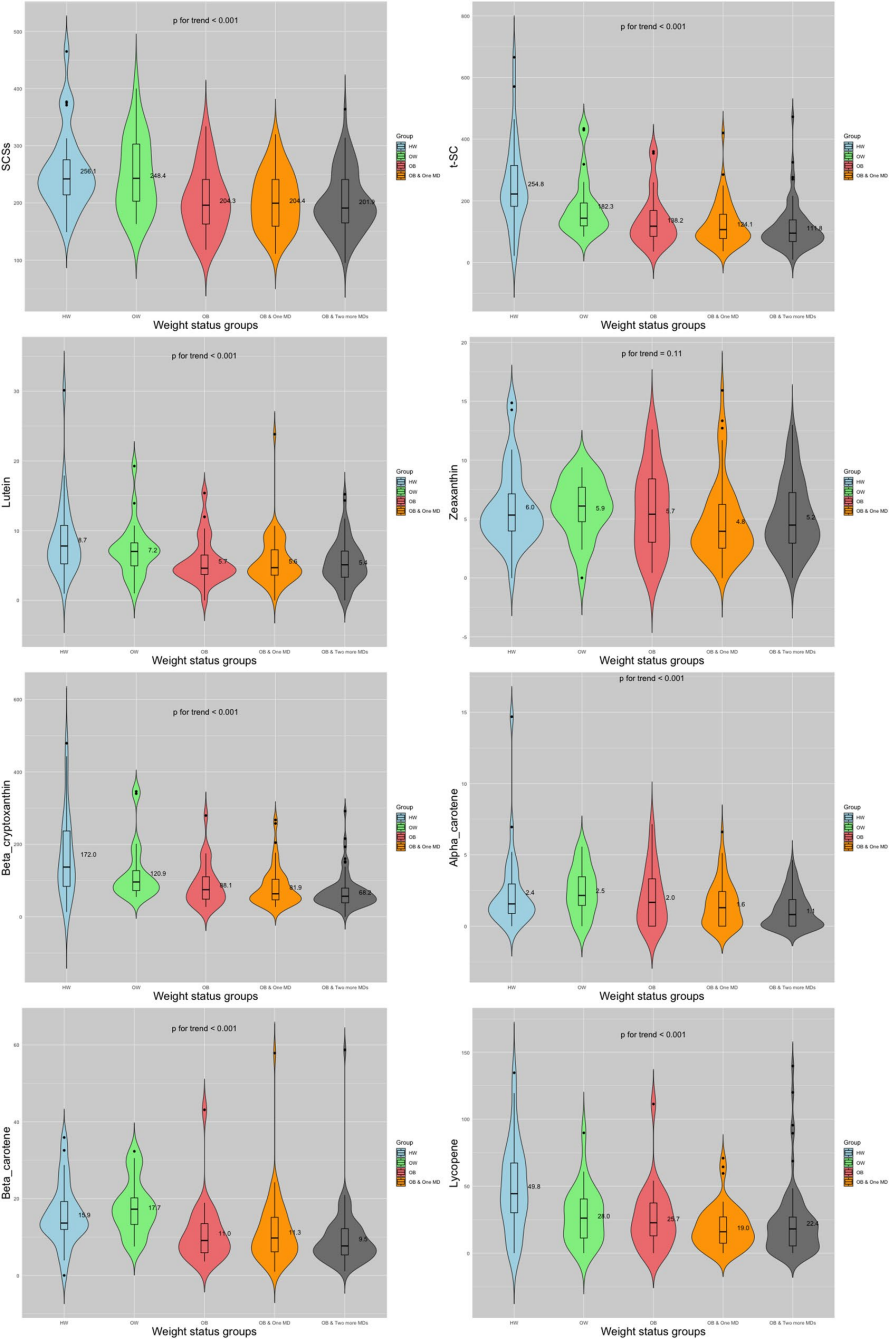
Trends in Skin Carotenoid Scores (SCSs), Total Serum Carotenoids (t-SC), and Six Individual Serum Carotenoids Across Weight Status GroupsThe violin plots display the distribution of SCSs, t-SC, and six individual serum carotenoids across five weight status groups: ‘HW’ (Healthy Weight), ‘OW’ (Overweight), ‘OB’ (Obesity), ‘OB & One MD’ (Obesity with one metabolic disorder), and ‘OB & Two or more MDs’ (Obesity with two or more metabolic disorders). The width of each violin represents the density of the data at various levels of the measured parameter, with wider areas indicating a higher concentration of data points.For each group, the dot shows the median value, the number shows the mean value (score for SCSs, μg/dL for serum carotenoids), the thick bar represents the interquartile range (IQR), and the thin lines indicate the data spread within 1.5 times the IQR, excluding outliers. Outliers, if present, are marked as individual points. The y-axis represents the levels of SCSs, t-SC, or carotenoids, while the x-axis denotes the weight status groups. Trends in the distribution highlight the variation in SCSs and serum carotenoid levels across different weight statuses.

As shown in Fig. 2, there is a decreasing trend in SCS, total serum carotenoids (t-SC), and individual carotenoids, including lycopene, beta-carotene, alpha-carotene, beta-cryptoxanthin, and lutein, across the five weight status groups: HW, OW, OB, OB & One MD, and OB & Two or more MDs (*p* for trend < 0.001). However, no such trend is observed for zeaxanthin (*p* for trend = 0.343).

### Correlation of SCS with t-SC and six individual serum carotenoids

**Fig. 3:**
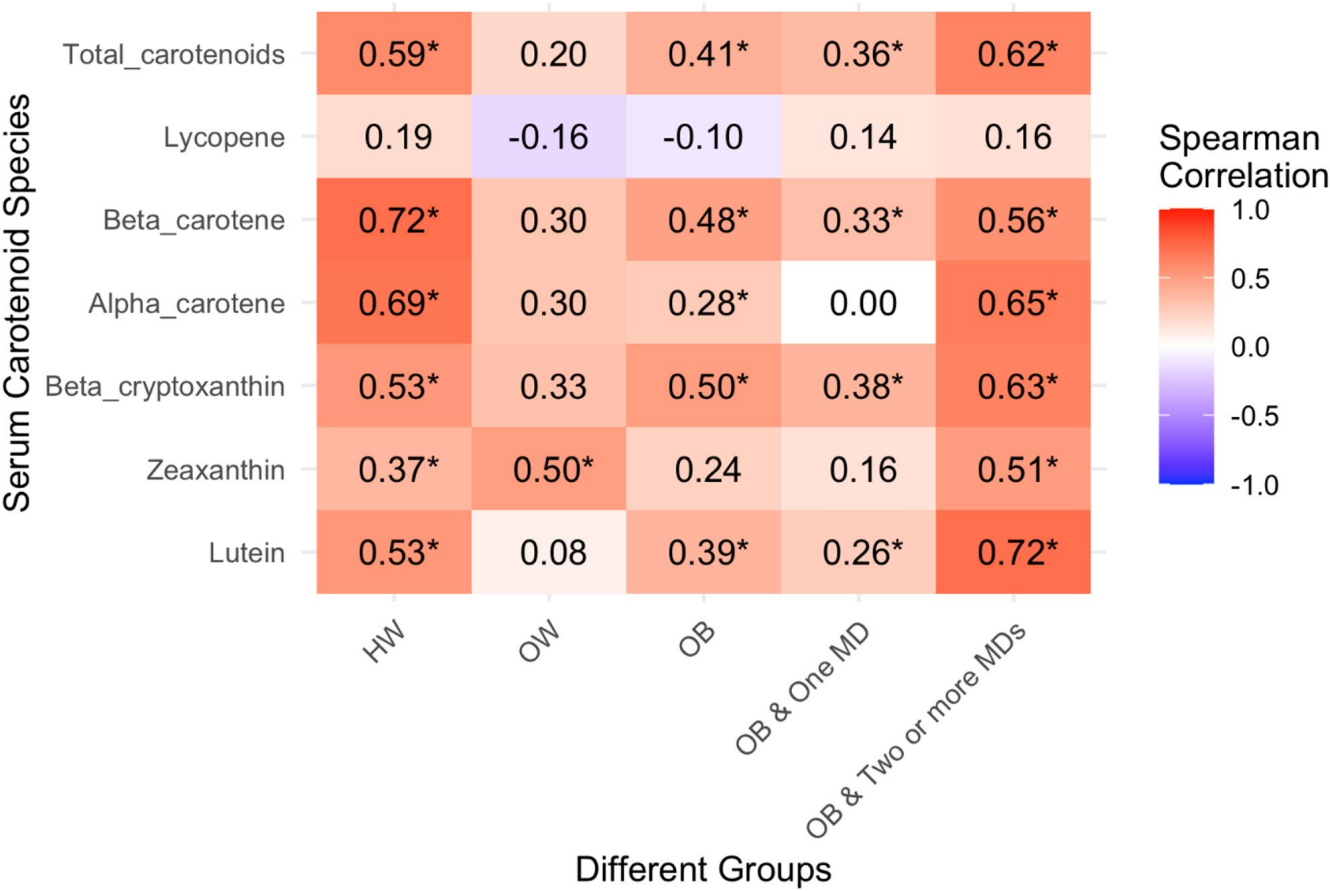
Heatmap of Spearman Correlations Between Serum Carotenoids and SCSs Across Weight GroupsThis figure shows the Spearman correlation coefficients between serum carotenoid levels and SCSs across five weight status groups: HW (Healthy Weight), OW (Overweight), OB (Obese), OB & One MD (Obese with one metabolic disorder), and OB & Two or more MDs (Obese with two or more metabolic disorders). The intensity of the red color indicates the strength of positive correlations, while the blue color represents negative correlations. An asterisk (*) denotes statistically significant correlations (p < 0.05).

As shown in Fig. 3, a general trend of positive correlations between serum carotenoids and SCS is observed across all groups. Significant positive correlations are consistently noted for total serum carotenoids (t-SC) and beta-carotene in nearly all groups, except for OW. Beta-cryptoxanthin and lutein display some variability, showing significant positive correlations in three groups but non-significant correlations in the HW and OW. Alpha-carotene correlates with SCS only in the HW and OB & Two or more MDs, while zeaxanthin is significant only in the OW and OB & Two or more MDs. Lycopene shows the weakest correlation with SCS, with no significant correlations in any of the five groups. In general, SCS correlates most with serum carotenoids in OB & Two or more MDs.

### Association of SCS with FVI and mediation by serum carotenoids

As shown in Table 2, after adjusting for age, sex, and supplement intake, a one-quartile increase in FVI was associated with a 20.86-unit increase in SCS among all participants (95% CI: 14.24, 27.48), a 45.98-unit increase among healthy participants (95% CI: 17.57, 74.38), and an 11.92-unit increase among those with obesity and two or more metabolic disorders (95% CI: 3.03, 20.81). Total serum carotenoids, beta-carotene, and beta-cryptoxanthin mediated the relationship between FVI and SCS by 22%, 15%, and 24%, respectively, among all participants. Total serum carotenoids mediated this relationship by 23% among healthy participants. Lycopene only showed correlation with SCS in the “OB with one MD” group.

**Table 2:** Coefficients (95%CI) for the change in SCSs associated with one-unit increase in FVI quartile, t-SC and individual serum carotenoids, stratified by weight status ^a^

β							
	Total		HW	OW	OB	OB with one MD	OB with two or more MDs
**FVI quartile**	**20.86 (14.24**,** 27.48)**		**45.98 (17.57**,** 74.38)**	12.99 (−13.49, 39.47)	12.20 (−11.27, 35.67)	10.81 (−2.24, 21.86)	**11.92 (3.03**,** 20.81)**
**t-SC (µg/100ml)**	**0.26 (0.19**,** 0.33)**		**0.25 (0.08**,** 0.41)**	**0.28 (0.08**,** 0.49)**	0.23 (−0.02, 0.48)	0.04 (−0.14, 0.22)	**0.22 (0.07**,** 0.37)**
*Prop. Mediated by t-SC* ^*b*^	**0.22 (0.08**,** 0.37)**		**0.23 (0.01**,** 0.56)**	0.84 (−5.09, 4.38)	0.44 (−3.39, 2.68)	−0.03 (−0.83, 0.27)	0.03 (−0.67, 0.37)
**Individual serum carotenoids**							
Lycopene (µg/100 ml)	0.44 (0.15, 0.73)		0.53 (−0.30, 1.36)	0.05 (−1.13, 1.22)	0.33 (−0.60, 1.26)	**−0.67 (−1.33**,** −0.01)**	0.29 (−0.10, 0.69)
*Prop. Mediated by lycopene* ^*b*^	0.02 (−0.06, 0.11)		−0.07 (−0.52, 0.19)	0.00 (−0.01, 1.35)	0.13 (−1.64, 2.92)	0.16 (−0.37, 1.19)	−0.03 (−0.35, 0.23)
Beta-carotene (µg/100 ml)	**2.91 (2.00**,** 3.83)**		**6.55 (3.78**,** 9.29)**	2.72 (−0.57, 6.01)	**2.84 (0.48**,** 5.20)**	0.03 (−1.50, 1.56)	**1.78 (0.33**,** 3.23)**
*Prop. Mediated by beta-carotene* ^*b*^	**0.15 (0.05**,** 0.28)**		0.30 (−0.09, 0.77)	−0.01 (−3.15, 3.60)	1.04 (−5.74, 5.44)	−0.02 (−0.51, 0.36)	−0.04 (−0.69, 0.12)
Alpha-carotene (µg/100 ml)	**8.85 (4.69**,** 13.01)**		**10.79 (2.04**,** 19.54)**	10.70 (−5.73, 27.12)	**9.19 (0.36**,** 18.02)**	7.45 (−0.05, 14.95)	2.31 (−6.58,11.18)
*Prop. Mediated by alpha-carotene* ^*b*^	0.05 (−0.03, 0.17)		0.07 (−0.27, 0.66)	−0.49 (−7.17, 5.77)	0.33 (−1.71, 1.81)	0.22 (−0.13, 1.34)	0.01 (−0.16, 0.14)
Beta-cryptoxanthin (µg/100 ml)	**0.34 (0.25**,** 0.43)**		**0.26 (0.06**,** 0.47)**	**0.36 (0.13**,** 0.60)**	0.32 (−0.04, 0.69)	0.13 (−0.10, 0.37)	**0.33 (0.10**,** 0.57)**
*Prop. Mediated by beta-cryptoxanthin* ^*b*^		**0.24 (0.10**,** 0.39)**	0.21 (−0.06, 0.59)	1.10 (−7.09, 4.63)	0.26 (−2.15, 1.91)	−0.07 (−0.72, 0.24)	0.06 (−0.38, 0.39)
Lutein (µg/100 ml)	**4.63 (2.36**,** 6.89)**		**6.39 (1.86**,** 10.92)**	**11.23 (5.90**,** 16.56)**	4.17 (−1.89, 10.23)	−0.14 (−3.61, 3.34)	**3.92 (0.74**,** 7.22)**
*Prop. Mediated by lutein* ^*b*^	0.06 (−0.05, 0.19)		0.21 (−0.02, 0.46)	0.94 (−2.81, 4.87)	0.42 (−3.05, 2.87)	−0.00 (−0.25, 0.28)	0.13 (−0.06, 0.66)
Zeaxanthin (µg/100 ml)	**4.78 (2.49**,** 7.07)**		5.49 (−2.40, 13.38)	3.43 (−7.45, 13.30)	5.55 (−1.85, 12.95)	1.40 (−2.07, 4.87)	**3.81 (0.60**,** 7.02)**
*Prop. Mediated by zeaxanthin* ^*b*^	0.02 (−0.07, 0.12)		0.05 (−0.18, 0.28)	−0.13 (−3.53, 3.27)	0.43 (−6.31, 4.38)	−0.07 (−0.61, 0.14)	−0.02 (−0.56, 0.27)

CI, Confidence interval; SCS: skin carotenoid score; FVI, fruit and vegetable intake; t-SC: total serum carotenoids; HW, healthy weight; OW, overweight; OB, obesity; MD, metabolic disorder

^a^ Adjusted for age, sex, and carotenoid supplement intake

^b^Prop. Mediated refers to the proportion of the total effect of FVI quartiles on SCSs that is explained by t-SC and individual serum carotenoids. It is calculated by dividing the indirect effect (the effect of FVI quartiles on SCSs through the mediator) by the total effect. For example, a Prop. Mediated value of 0.01 indicates that 1% of the total effect is mediated by the mediator

### Association of SCS with inflammatory markers

**Fig. 4:**
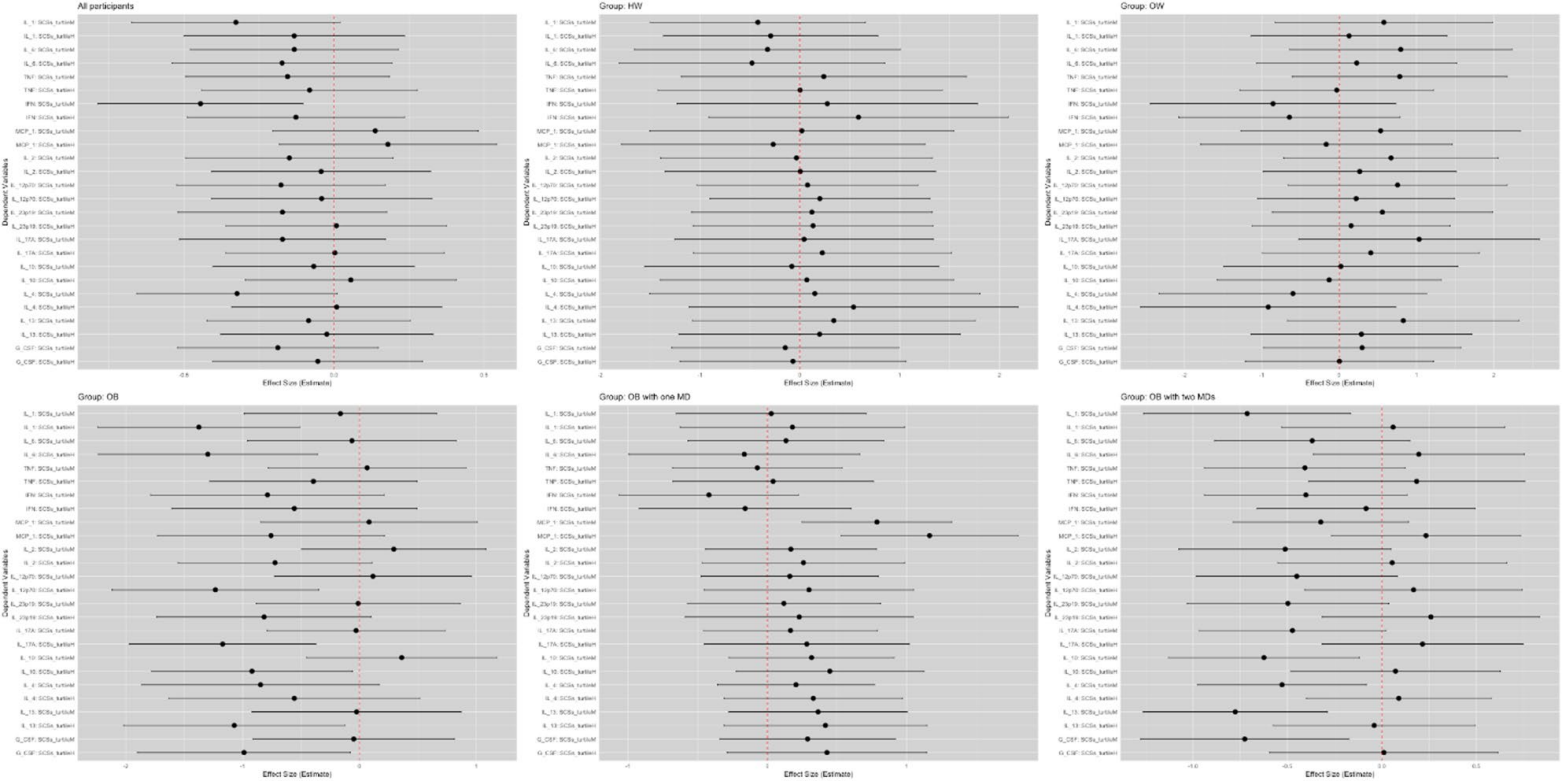
Forest Plot of SCSs tertiles Across Different Groups and Inflammatory MarkersThis figure presents the effect size estimates and 95% confidence intervals for the association between SCSs (categorized into different tertiles) and multiple inflammatory markers (IL-1, IL-6, TNF, IFN, MCP-1, IL-2, IL-12p70, IL-23p19, IL-17A, etc.), stratified by participant groups: All participants, HW (Healthy Weight), OW (Overweight), OB (Obese), OB with one MD (Metabolic Disorder), and OB with two MDs. Y-axis: Each row represents the regression coefficient (effect size) for a specific inflammatory marker, with separate lines corresponding to different categories of SCSs tertile. X-axis: The effect size estimates from the linear regression models, adjusted for sex, age, and BMI. The dashed red vertical line at x = 0 indicates no effect. Error Bars: The horizontal lines represent the 95% confidence intervals for the effect sizes. The different panels represent the stratified analysis by weight categories, allowing comparison of the relationships between SCSs and inflammatory markers in various health conditions.

Figure 4 shows the associations between SCS and various inflammatory markers, stratified by weight group. Among all participants, SCS is negatively associated with IFN. In healthy participants and those who are overweight, no significant associations were found between SCS and inflammatory markers. In the obese group, SCS is negatively associated with IL-1, IL-6, IL-12p70, IL-17 A, IL-10, IL-13, and G-CSF. Among participants with obesity and one metabolic disorder, SCS is positively associated with MCP-1. For those with obesity and two or more metabolic disorders, SCS is negatively associated with IL-1, IL-10, IL-4, IL-13, and G-CSF.

## Discussion

This study validates the use of RS-based SCS in children and adolescents across varying obesity levels and metabolic disorders. Healthy children and adolescents averaged an SCS of 256.1 ± 69.9, with scores decreasing as obesity and metabolic disorders increased, paralleling trends in serum carotenoid levels, especially lutein. Except for lycopene, SCS correlated with total serum carotenoids and all individual carotenoids, with beta-carotene and lutein showing the strongest correlations in healthy and severely obese groups, respectively. Associations between SCS and inflammatory markers were inconsistent.

SCS measurements facilitate international comparisons. This study reports Chinese children and adolescents’ SCS ranging from 201.9 ± 53.3 to 256.1 ± 69.9, suggesting a generally lower range than American and Japanese data. A systematic review reported SCS between 156.2 and 380 among American children and adolescents [30], while a study on Japanese children and adolescents found an average SCS of 366.8 [40]. This disparity may be partly attributable to distinct dietary patterns. Compared to western diets rich in diverse carotenoid sources and the Japanese diet with high seafood-derived astaxanthin, traditional Chinese diets in many regions are heavily centered on cooked vegetables and staple grains, where cooking methods and limited fat intake can significantly reduce carotenoid bioavailability. Findings highlight a clear descending trend in SCS with increasing weight and metabolic disorders, consistent with studies linking SCS to BMI and body fat [8, 41–44]. These results bolster the construct validity of SCS, showing their inverse relationship with weight status and serum carotenoids.

The descending SCS trend mirrors serum carotenoid levels except for zeaxanthin, reflecting dietary variations. Notably, SCS align more closely with lutein, whose distribution differs from zeaxanthin due to dietary sources. Prior studies predominantly focus on broad populations, with reported correlations between VM^®^ SCS and serum carotenoids ranging from 0.54 to 0.87 [19–22, 31]. This study adds evidence in Chinese children and adolescents, finding moderate to strong correlations in healthy and severely obese groups, while weaker correlations in overweight and moderately obese groups may stem from dietary variability and lipid metabolism.

Lycopene’s weak SCS correlation aligns with previous studies [17, 20, 43]. The weak correlation can be attributed to two primary factors [45]. Firstly, the reflection spectroscopy method is optimized for the absorption spectra of carotenoids like lutein and β-carotene, which differs from that of lycopene. Secondly, lycopene may be deposited more deeply in the dermis, beyond the optimal detection depth of the light signal, thereby attenuating its measured contribution to the total SCS. SCS’ strong correlations with beta-carotene in healthy groups and lutein in severely obese groups suggest their potential as specific dietary biomarkers. SCS reflect FVI among Chinese children and adolescents, with each quartile increase in FVI corresponding to a 20.86-point rise in SCS. Carotenoids accumulate in the skin via subcutaneous fat, blood, and glandular secretion [46]. Total serum carotenoids, beta-carotene, and beta-cryptoxanthin mediate the FVI-SCS relationship, though mediation effects vary among carotenoids.

SCS were linked to inflammatory markers primarily in obese and metabolically disordered groups, supporting carotenoids’ antioxidant roles [47, 48]. Unexpected correlations, such as with IL-4 and MCP-1, suggest complex interactions between carotenoids and inflammation in obesity [49–51]. Further research is needed to elucidate these relationships and their implications for chronic inflammation [52, 53].

Strengths of this study include the comprehensive validation of VM^®^ SCS across obesity levels and metabolic disorders in Chinese children and adolescents. However, the data used in this study are not longitudinal, which prevents us from establishing causality. The control group (*n* = 30) is small compared to the other groups, which may reduce statistical power. Other limitations include reliance on FFQs and geographic specificity. Future studies should explore regional variations, gold-standard dietary methods, and longitudinal responsiveness of SCS to dietary interventions.

## Conclusion

The present study validated the use of RS based SCS in children and adolescents, demonstrating moderate correlations with FFQ-based FVI and total serum carotenoids. RS based SCS effectively distinguished between different weight status groups with metabolic disorders, aligning with serum carotenoid levels. However, it was less sensitive to lycopene intake, highlighting a limitation in measuring certain dietary patterns. The significant associations between SCS and inflammatory markers in metabolically disordered groups suggest its potential as a dual-purpose biomarker for both dietary intake and inflammation, offering a practical tool for assessing diet and health in children and adolescents.

## Supplementary Information

Supplementary Material 1

## Data Availability

The raw data that support the findings of the present study are available from the corresponding author upon request.

## Abbreviations

FVI: Fruit and Vegetable Intake
SCS: Skin Carotenoid Scores
IL: Interleukin
Veggie Meter^®^: TNF-α: Tumor Necrosis Factor-Alpha VM^®^
