# Supplementary material for: Skin carotenoids indicate diet, serum carotenoids, and inflammation across obesity and metabolic status in children
Source: Nutr Metab (Lond). 2026 Feb 22;23:41. doi: 10.1186/s12986-026-01075-7 (PMC13032390; doi:10.1186/s12986-026-01075-7)
Supplement: Supplementary file 1 — Supplementary Material 1 [file 12986_2026_1075_MOESM1_ESM.docx]

Figure S1. Participants flow chart
